# Inflammaging, immunosenescence, and cardiovascular aging: insights into long COVID implications

**DOI:** 10.3389/fcvm.2024.1384996

**Published:** 2024-06-26

**Authors:** Ludmila Müller, Svetlana Di Benedetto

**Affiliations:** Center for Lifespan Psychology, Max Planck Institute for Human Development, Berlin, Germany

**Keywords:** aging, immunosenescence, inflammaging, cardiovascular diseases, COVID-19, long COVID

## Abstract

Aging leads to physiological changes, including inflammaging—a chronic low-grade inflammatory state with significant implications for various physiological systems, particularly for cardiovascular health. Concurrently, immunosenescence—the age-related decline in immune function, exacerbates vulnerabilities to cardiovascular pathologies in older individuals. Examining the dynamic connections between immunosenescence, inflammation, and cardiovascular aging, this mini-review aims to disentangle some of these interactions for a better understanding of their complex interplay. In the context of cardiovascular aging, the chronic inflammatory state associated with inflammaging compromises vascular integrity and function, contributing to atherosclerosis, endothelial dysfunction, arterial stiffening, and hypertension. The aging immune system's decline amplifies oxidative stress, fostering an environment conducive to atherosclerotic plaque formation. Noteworthy inflammatory markers, such as the high-sensitivity C-reactive protein, interleukin-6, interleukin-1β, interleukin-18, and tumor necrosis factor-alpha emerge as key players in cardiovascular aging, triggering inflammatory signaling pathways and intensifying inflammaging and immunosenescence. In this review we aim to explore the molecular and cellular mechanisms underlying inflammaging and immunosenescence, shedding light on their nuanced contributions to cardiovascular diseases. Furthermore, we explore the reciprocal relationship between immunosenescence and inflammaging, revealing a self-reinforcing cycle that intensifies cardiovascular risks. This understanding opens avenues for potential therapeutic targets to break this cycle and mitigate cardiovascular dysfunction in aging individuals. Furthermore, we address the implications of Long COVID, introducing an additional layer of complexity to the relationship between aging, immunosenescence, inflammaging, and cardiovascular health. Our review aims to stimulate continued exploration and advance our understanding within the realm of aging and cardiovascular health.

## 1 Introduction

As we age, our bodies embark on a remarkable journey of physiological changes, collectively known as aging (1–3). Inflammaging, a term conceived to describe the chronic low-grade inflammation associated with aging (4), has emerged as a fundamental factor in the intricate landscape of both aging and age-related diseases. Characterized by a persistent and low-level inflammatory state, inflammaging implies a complex interplay of molecular and cellular events that contribute to the overall aging process. The implications of inflammaging extend beyond chronological aging, encompassing its profound impact on various physiological systems (5). Of particular interest is its intricate relationship with cardiovascular health, as increasing evidence suggests that inflammaging plays a significant role in the development and progression of cardiovascular diseases (6, 7).

Concurrently, immunosenescence, the age-related decline in immune function, represents another aspect of the aging process with direct implications for general health deteriorations observed in aging populations (1, 8, 9). The aging immune system undergoes significant transformations, which detrimentally affect its capacity to generate robust immune responses against both external and internal challenges, thereby increasing susceptibility to a range of diseases (10, 11). Within the context of cardiovascular aging, the implications of immunosenescence become especially noteworthy, contributing to the heightened vulnerability to cardiovascular pathologies observed in older individuals.

In the following sections we aim to dissect the multifaceted dimensions and the dynamic connections between inflammaging, immunosenescence, and cardiovascular aging. By understanding the intricate molecular and cellular mechanisms underlying these phenomena, we aim to unravel their collective influence on cardiovascular health. Additionally, the relevance of these phenomena gains particular significance in the context of emerging health challenges, such as Long COVID. The complex interplay among the immunosenescence, inflammaging, and cardiovascular health following COVID-19 presents a multidimensional puzzle that requires in-depth exploration to gain holistic insights into these processes. By unraveling their relationships, we may pave the way for developing strategies aimed at addressing age-associated immune alterations, cardiovascular dysregulation and post-COVID complications. These strategies could significantly enhance the overall quality of life for the elderly.

## 2 Inflammaging and cardiovascular diseases

**Figure 1:**
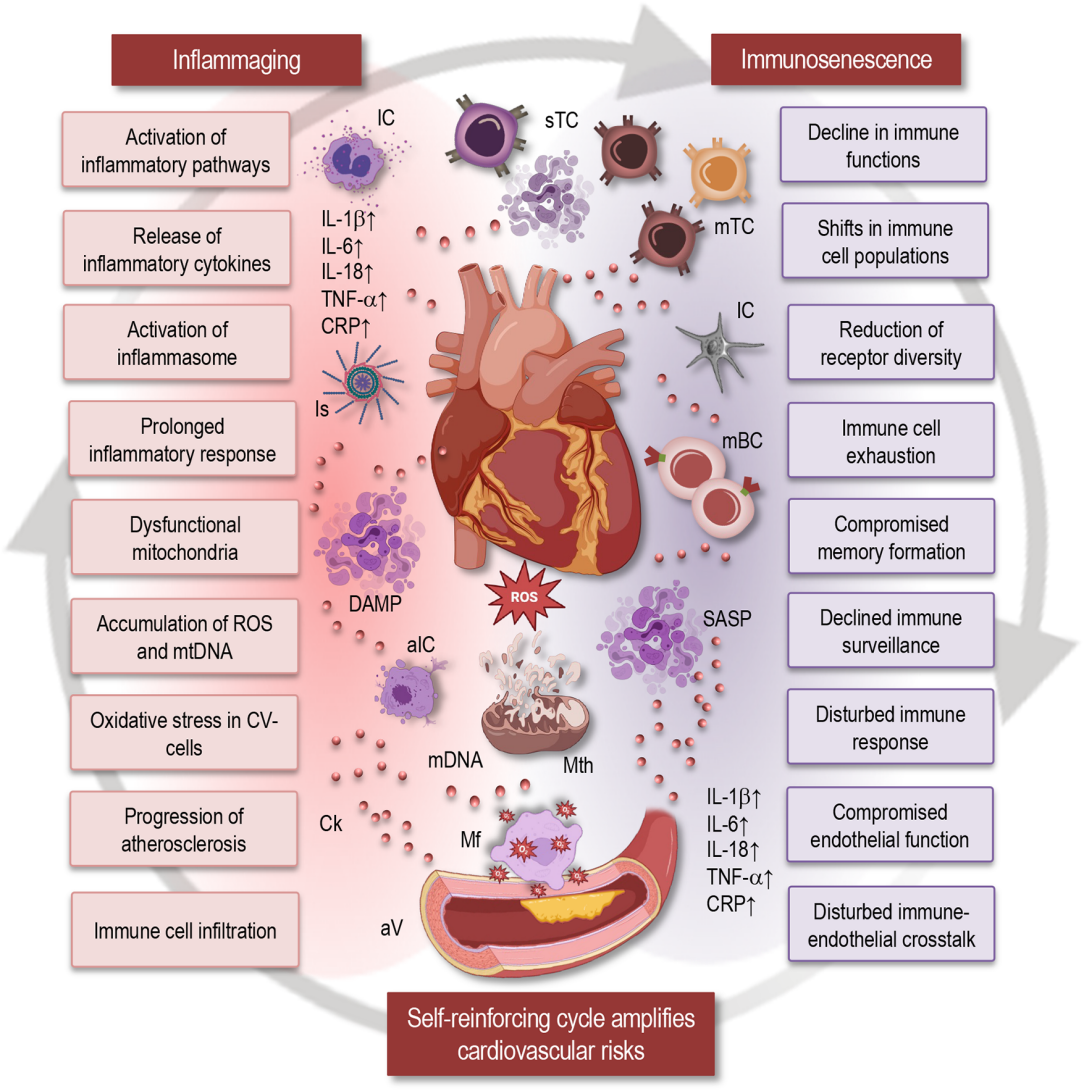
Impact of inflammaging and immunosenescence on cardiovascular health. This simplified scheme illustrates how inflammaging and immunosenescence contribute to cardiovascular aging, highlighting their reciprocal relationship and implications for cardiovascular health. Inflammaging: Chronic low-grade inflammation associated with aging is characterized by the release of inflammatory cytokines (IL-1β, IL-6, IL-18, TNF-α, CRP), prolonged inflammatory responses, activation of the inflammasome, and inflammatory pathways. Dysfunctional mitochondria and the accumulation of reactive oxygen species (ROS) and mitochondrial DNA (mtDNA) contribute to oxidative stress in cardiovascular cells, promoting the progression of atherosclerosis. Immunosenescence: Aging leads to shifts in immune cell populations, resulting in a decline in immune functions. Key features include reduced receptor diversity, immune cell exhaustion, compromised memory formation, and declined immune surveillance. This ultimately disturbs the immune response and compromises endothelial function. Self-reinforcing cycle: The cycle of immunosenescence and inflammaging amplifies cardiovascular risks through immune cell infiltration, disturbed immune-endothelial crosstalk, and compromised endothelial function. The interaction between inflammaging and immunosenescence forms a self-reinforcing cycle that exacerbates cardiovascular dysfunction. IC, immune cells; Is, inflammasome; sTC, senescent T cells; mTC, memory T cells; mBC, memory B cells; SASP, senescence-associated secretory phenotype; DAMP, damage-associated molecular patterns; aIC, activated immune cells; Ck, cytokine; Mth, mitochondria; Mf, macrophage; ROS, reactive oxygen species; aV, atherosclerotic vessel; mtDNA, mitochondrial DNA; CV, cardiovascular; IL, interleukin; TNF, tumor necrosis factor; CRP, C-reactive protein.

In the complex interplay between aging and cardiovascular health, inflammaging emerges as a central player, orchestrating molecular and cellular events that significantly impact the development and progression of cardiovascular diseases (6, 12, 13). A multidimensional dynamic interaction, involving cytokines, inflammasomes, and senescent cells, actively triggering a prolonged pro-inflammatory environment (Figure 1), characterize age-associated immune changes (7). These shifts in immune dynamics and inflammatory responses during aging hold significant consequences, with one notable outcome being the heightened susceptibility to cardiovascular diseases among the elderly (14). Elucidating the molecular and cellular mechanisms underlying this complex relationship provides essential insights into the development and progression of cardiovascular pathologies.

At the core of the inflammaging's profound influence on cardiovascular health lies the sophisticated orchestration of chronic inflammatory signaling pathways. The persistent release of pro-inflammatory cytokines, such as interleukin-6 (IL-6), IL-1β, and tumor necrosis factor-alpha (TNF-α), triggers inflammatory signaling pathways that compromise vascular integrity and function. TNF-α activates several key signaling pathways (15–19), including the nuclear factor (NF)-κB, the c-Jun NH2-terminal kinase (JNK), and the p38 mitogen-activated protein kinase (MAPK). Activation of NF-κB leads to increased expression of pro-inflammatory cytokines, adhesion molecules, and chemokines. This promotes endothelial cell activation and leukocyte adhesion, contributing to vascular inflammation and endothelial dysfunction. JNK activation induces the expression of inflammatory mediators and apoptotic signals, which can lead to endothelial cell apoptosis and a compromised endothelial barrier, impairing vascular integrity. Activation of p38 MAPK results in the production of pro-inflammatory cytokines and stress responses. This enhances inflammation and oxidative stress within the vascular endothelium, further damaging the vascular structure and function. Collectively, these pathways lead to increased vascular permeability, inflammation, and oxidative stress, ultimately compromising vascular integrity and contributing to various cardiovascular diseases.

Additionally, NF-κB functions as a central mediator in the stimulation of pro-inflammatory genes, and the upregulation of the NF-κB pathway has been consistently observed in inflammatory disorders associated with aging (20–22). The activation of the NF-κB-mediated inflammatory response is markedly driven by the formation of a Nod-like receptor pyrin domain containing 3 (NLRP3) inflammasome. Enhanced NLRP3 inflammasome activity is linked to age-related pathological conditions such as atherosclerosis, type 2 diabetes, and Alzheimer's disease (23). Increased NLRP3 inflammasome activity in aging leads to heightened oxidative stress and mitochondrial dysfunction, further promoting cellular and tissue damage (Figure 1). Moreover, persistent NLRP3 activation contributes to immunosenescence, reducing the immune system's ability to respond to infections and clear damaged cells, thereby exacerbating age-related conditions. NLRP3 activation triggers the production of pro-inflammatory cytokines IL-1β and IL-18. This chronic inflammation contributes to diseases such as atherosclerosis, type 2 diabetes, and Alzheimer's disease (24, 25). Among the various downstream pro-inflammatory pathways activated by the NLRP3 inflammasome, IL-1β and IL-18 emerge as potential contributors to inflammaging. It is noteworthy that the expression of IL-18 increases during the aging process in humans (12). Researchers found that the mRNA of the pro-inflammatory cytokine IL-18 was upregulated in aged female hearts but not in male hearts, indicating a gender-specific inflammatory response (26).

The aging of the heart tissue is linked to a general decline in the mitochondrial function and the accumulation of dysfunctional mitochondria (Figure 1), primarily attributed to the dysregulation of quality control processes (12, 27). Mitochondria play a crucial role as a source of reactive oxygen species (ROS), serving both as signaling molecules and potential initiators of harm (Figure 1). The accumulation of ROS contributes to heightened oxidative stress, fostering the subsequent buildup of damaged DNA, proteins, lipids, as well as mitochondrial DNA (mtDNA) damage and release. In the elderly, there is a substantial increase in the release of mtDNA from the mitochondrial matrix into the cell cytosol, triggering the generation of damage-associated molecular patterns (DAMPs). DAMPs are molecules released by stressed or dying cells that trigger an immune response by signaling tissue damage. This process is considered a catalyst for inflammatory responses (28, 29).

The age-related chronic inflammatory state associated with inflammaging induces oxidative stress within the vascular endothelium. ROS generated under these conditions lead to endothelial dysfunction, compromising the normal physiological functions of blood vessels (Figure 1). Endothelial dysfunction, in turn, plays a crucial role in the initiation and progression of atherosclerosis, contributing to the development of cardiovascular diseases. Namely, endothelial dysfunction initiates atherosclerosis by impairing the normal function of the endothelium, leading to reduced nitric oxide bioavailability, increased permeability to lipoproteins, and enhanced expression of adhesion molecules. This promotes the adherence and infiltration of inflammatory cells into the arterial wall, fostering a chronic inflammatory environment that contributes to plaque formation and progression (30, 31). Beyond atherosclerosis, inflammaging influences vascular remodeling processes. The persistent low-grade inflammation stimulates the dysregulation of vascular smooth muscle cells, promoting their proliferation and migration. This dysregulation contributes to arterial stiffening, a hallmark of vascular aging, and plays a role in the development of hypertension and other cardiovascular pathologies (13, 30, 32).

Activation of immune cells, particularly monocytes, macrophages, and T lymphocytes, within the vascular microenvironment further promotes oxidative stress by increasing the production of ROS. This oxidative stress damages cellular components, promotes inflammation, and facilitates pathophysiological changes like endothelial dysfunction, smooth muscle cell proliferation, and atherosclerotic plaque formation (Figure 1). In response to the persistent inflammatory stimuli, these activated immune cells may infiltrate the vascular walls. Once present, they contribute to oxidative stress, fostering an environment conducive to atherosclerotic plaque formation. Oxidative stress promotes atherosclerotic plaque formation by damaging endothelial cells, increasing the oxidation of low-density lipoprotein (LDL), and fostering an inflammatory environment. This leads to the recruitment of immune cells and the buildup of fatty deposits in arterial walls. Macrophages, for instance, uptake oxidized low-density lipoproteins, transforming into foam cells that contribute to the formation of atherosclerotic lesions (33, 34). They accumulate within the arterial walls and contribute to the formation and growth of atherosclerotic plaques, which can obstruct blood flow and lead to cardiovascular diseases.

Several inflammatory markers take center stage in the context of cardiovascular aging. The high-sensitivity C-reactive protein (hsCRP), a notable marker, stands out as a systemic indicator of inflammation and is closely associated with an elevated risk of cardiovascular events. Elevated levels of hsCRP indicate chronic low-grade inflammation, which is a key factor in the development and progression of atherosclerosis. This persistent inflammation can lead to the formation and rupture of atherosclerotic plaques, increasing the risk of heart attacks and strokes (31, 35, 36). Additionally, IL-1β, IL-6, and IL-18, prominent members of the inflammatory milieu, have been implicated in the pathogenesis of cardiovascular diseases by promoting inflammation within the vascular system. IL-1β contributes to the formation and instability of atherosclerotic plaques, IL-6 is involved in endothelial dysfunction and the systemic inflammatory response, and IL-18 exacerbates vascular inflammation and plaque instability, collectively leading to an increased risk of cardiovascular events. These inflammatory mediators provide key insights into the intricate interplay between inflammation and cardiovascular aging (7, 12, 13).

In summary, the exploration of complex molecular and cellular mechanisms attributed to inflammaging uncovers the central role of this low-grade inflammation state in shaping the landscape of cardiovascular diseases during aging. From the activation of proinflammatory signaling pathways to the highlighting of specific inflammatory markers associated with cardiovascular aging, research has demonstrated a nuanced array of molecular and cellular mechanisms, each contributing to the multifaceted pathophysiology observed in aging individuals. However, it is essential to recognize that these age-related changes do not unfold in isolation; rather, they are part of a simultaneous process involving the aging of the immune system, known as immunosenescence. In the next section, we will unravel the multidimensional relationship between immunosenescence and cardiovascular dysfunction, shedding light on how the age-related decline in immune function contributes to the complex tapestry of cardiovascular pathologies during aging.

## 3 Immunosenescence and cardiovascular dysfunction

Immunosenescence summarizes a series of multilayered age-related changes of the immune system that collectively impact immune function over time (37, 38). This phenomenon influences a myriad of cellular and molecular processes and reflects a persistent alteration in the immune response dynamics, resulting in a less effective and coordinated defense against internal and external challenges. Key characteristics of immunosenescence include shifts in immune cell populations, functional alterations, and changed signaling pathways. Immune checkpoints—crucial regulators of immune responses—also experience functional decline during immune aging (39). Inhibitory receptors such as Programmed Cell Death Protein 1 (PD-1) and Cytotoxic T-Lymphocyte Associated Protein 4 (CTLA-4) become upregulated, dampening T-cell activity. The dysregulation of these checkpoints results in a skewed balance of immune activity, contributing to the impairment of immune responses within the cardiovascular microenvironment. All of these changes collectively contribute to the overarching decline in immune competence as individuals age (40, 41), creating a milieu that fosters an increased susceptibility to cardiovascular diseases (42, 43).

A characteristic feature of immunosenescence is the reconfiguration of immune cell populations, crucially influencing the orchestration of immune responses (38, 41, 44). During the aging process, cells of adaptive immunity undergo remodeling (Figure 1) marked by a reduction in receptor diversity and the compromised formation of immunological memory (45–49). Moreover, in the elderly, peripheral T cells commonly demonstrate diminished absolute numbers, a skewed CD4:CD8 ratio, and an accumulation of terminally differentiated effector memory T cells, which no longer proliferate but remain in the circulation. Lifelong antigen exposure, decreased thymic output, replicative senescence, altered homeostasis, and chronic inflammation collectively result in a T-cell population skewed towards terminal differentiation (50, 51). These T cells have a diminished proliferative capacity due to a combination of telomere shortening, cell cycle arrest, altered signaling, metabolic shifts, epigenetic changes, and the adoption of a senescence-associated secretory phenotype (SASP), all contributing to their reduced ability to expand in response to new antigens (52–54). The compromised adaptive immunity not only affects the immediate defense against pathogens or damaged cells but also hinders the resolution of inflammation, hence fostering a chronic inflammatory state within the cardiovascular microenvironment (6, 55).

With advancing age, T lymphocytes undergo a progressive impairment in their functional ability (10, 48, 49). Prolonged exposure to antigens and chronic inflammatory stimuli lead to a state where these immune cells, especially CD8^+^ cytotoxic T cells, lose their effector functions. The altered capacity to produce cytokines and cytotoxic molecules, and to respond to new challenges leaves the immune system less equipped to counteract cardiovascular insults (42, 56). The consequence of such T-cell exhaustion is a compromised immune surveillance within the cardiovascular system (56, 57). The surveillance, crucial for identifying and eliminating potentially harmful cells, becomes less efficient. This reduced vigilance contributes to an environment where cellular stress, damage, and potential threats within the vasculature go unchecked, hence fostering conditions conducive to the initiation and progression of cardiovascular diseases (58).

The complex relationship between immune aging and cardiovascular dysfunction extends to causing profound implications for vascular homeostasis. The cascading effects of immunosenescence play a pivotal role in shaping the landscape of the vasculature during aging (59). Endothelial cells, which form the inner lining of blood vessels, are essential for regulating vascular tone, blood flow, and the recruitment of immune cells. In the context of age-related immune exhaustion, these endothelial cells experience dysfunction due to a confluence of factors, including oxidative stress and reduced nitric oxide bioavailability (30, 60). Endothelial cell dysfunction involves impaired nitric oxide production, increased expression of adhesion molecules, enhanced permeability to lipoproteins, and decreased anti-inflammatory and anti-thrombotic properties, ultimately contributing to vascular inflammation and atherosclerosis (30, 60, 61). The compromised endothelial function also disrupts the delicate balance of vasoconstriction and vasodilation, thus contributing to arterial stiffness and hypertension.

Effective immune-endothelial crosstalk is critical for maintaining vascular homeostasis. Immune cells, particularly T lymphocytes, monocytes, and macrophages actively communicate with endothelial cells to regulate inflammation and repair processes (33, 62–64). However, immune aging disrupts this delicate balance. Impaired immune responses hinder the resolution of inflammation, perpetuating a pro-atherogenic environment within the vasculature (Figure 1). Additionally, dysfunctional endothelial cells struggle to orchestrate proper immune cell recruitment and activation, further exacerbating the compromised vascular homeostasis (12). Moreover, dysfunctional endothelial cells facilitate the entry of immune cells into the vessel wall, thus contributing to the formation of atherosclerotic plaques (33). As immune responses become inefficient, the resolution of inflammation within these plaques is impaired, perpetuating a state of chronic vascular inflammation.

Vascular homeostasis relies on efficient repair mechanisms to counteract cellular stress and damage. Examples of immune cells involved in tissue repair include macrophages, which phagocytose debris and promote tissue remodeling, and T lymphocytes, which release cytokines that stimulate tissue regeneration and repair processes. However, the profound consequences of immunosenescence hinders these repair processes (59, 60, 64, 65). The diminished functionality of immune cells, especially those involved in tissue repair, compromises the ability to restore vascular integrity. This impairment further contributes to the cumulative damage within the vasculature, hence fostering an environment where dysfunctional endothelial and immune cells create a self-perpetuating cycle of vascular compromise (59, 66, 67).

Thus, the age-related decline in immune function sets the stage for an altered immune landscape. The reduced functionality of T lymphocytes, impaired adaptive immunity, and compromised immune surveillance collectively contribute to an immune system that is less equipped to counteract cardiovascular challenges. This decline in immune competence creates an environment where the resolution of inflammation becomes less efficient, hence further promoting a pro-inflammatory milieu within the cardiovascular system. Understanding the nuanced dynamics between immunosenescence, inflammaging and their cumulative impact on cardiovascular dysfunction might provide critical insights into the vulnerabilities of the aging cardiovascular system.

## 4 Interplay of immunosenescence with inflammaging in cardiovascular dysfunction

The intersection of immunosenescence and inflammaging uncovers a complex interplay that significantly contributes to cardiovascular dysfunction during the aging process (13, 32). Both phenomena, characterized by alterations in the immune system and chronic low-grade inflammation, converge to shape the complex landscape of age-associated cardiovascular pathologies (32, 33, 59). Moreover, the reciprocal relationships between immunosenescence and inflammaging create a dynamic interplay that profoundly influences the setting of cardiovascular dysfunction during aging (Figure 1). The compromised immune responses characteristic of immunosenescence play a pivotal role in amplifying the chronic inflammatory state associated with inflammaging. With diminished immune surveillance and impaired adaptive immunity, the resolution of inflammation becomes less efficient. This inefficiency contributes to the persistence of inflammatory signals within the cardiovascular microenvironment, hence boosting a pro-inflammatory milieu that accelerates the progression of cardiovascular diseases (7, 12, 68).

Conversely, the persistent inflammatory signals from inflammaging exacerbate the functional decline of immune cells, thus creating a self-reinforcing cycle. Pro-inflammatory cytokines actively contribute to the accelerated immunosenescent phenotype. These cytokines not only directly impact the immune cell functionality by modulating their activation, proliferation, differentiation, and effector functions but also disrupt the delicate balance of the immune system by skewing immune responses towards inflammation or suppression, leading to autoimmune diseases, chronic inflammation, or immunodeficiency (69). Such disbalances further compromise the ability of the immune system to mount effective responses against cardiovascular challenges.

One of the central aspects of the reciprocal relationship lies in its impact on the immune cell repertoire. Immunosenescence, marked by a reduction in receptor diversity, limits the specificity and adaptability of the immune response (37). This reduction, coupled with the chronic inflammatory signals from inflammaging, further restricts the ability of immune cells to recognize and respond to emerging threats (such as pathogenic microorganisms, oxidative stress, lipid accumulation, cellular damage, and autoimmune reactions) within the cardiovascular microenvironment (12, 13, 32, 68). The resultant compromised immune cell repertoire becomes a hallmark of the reciprocal interplay, thus contributing to the susceptibility to cardiovascular diseases. Therefore, unraveling the reciprocal relationship between immunosenescence and inflammaging provides critical insights into potential therapeutic targets. Strategies aimed at breaking this self-reinforcing cycle may involve interventions to rejuvenate the immune function (70–78), modulate chronic inflammation (79–83), or disrupt specific molecular pathways (80, 84, 85) that contribute to the reciprocal amplification. Examples of interventions to rejuvenate immune function include caloric restriction, exercise, pharmacological agents like rapamycin, and potential therapies such as thymic rejuvenation and adoptive transfer of young immune cells (76, 82, 86).

Taken together, the complex reciprocal relationships create a self-reinforcing cycle where immunosenescence and inflammaging synergistically drive the progression of cardiovascular dysfunction. As immunosenescence compromises the immune function, chronic inflammation persists and intensifies. Simultaneously, the inflammatory milieu accelerates the decline in immune function, perpetuating a cycle that continuously amplifies the vulnerabilities of the aging cardiovascular system. By targeting these reciprocal interactions, novel therapeutic approaches may emerge to alleviate a cardiovascular dysfunction and promote healthy aging in the elderly population.

## 5 Insights into long COVID implications

In recent years, in addition to the already complex impact of immunosenescence and inflammaging on cardiovascular health in the elderly, a new player has entered this complex interplay—COVID-19. This viral infection has disproportionately affected older individuals, introducing an additional layer of complexity to the already complicated relationship between aging and cardiovascular health. Moreover, as we navigate the evolving landscape of health challenges in the elderly, it is crucial to recognize the emergence of Long COVID—a condition that extends beyond acute infection and presents potential long-term consequences, further complicating the nuanced interplay between aging, immunosenescence, inflammaging, and cardiovascular health.

Long COVID, or post-acute sequelae of SARS-CoV-2 infection, is defined as a multisystemic condition of persistent symptoms and is characterized by its complexity and insufficiently understood nature. It impacts individuals who have recovered from the acute phase of COVID-19, but who are still experiencing a variety of symptoms for weeks or even months thereafter (87, 88). This syndrome extends its impact across multiple organ systems, with cardiovascular complications often taking center stage as the most prominent features. The range of symptoms encompass, but are not limited to, chest pain, fatigue, heart failure, myocardial injury, arrhythmias, vascular injury, thrombosis, and dysautonomia (89, 90).

In a group of 150,000 post-COVID veterans, researchers revealed that even a mild case of COVID-19 can heighten an individual's risk of having cardiovascular problems for at least a year post-diagnosis (91). Rates of various conditions, including heart failure and stroke, were markedly higher in individuals who had recovered from COVID-19 compared to similar counterparts who had not contracted the disease. Notably, this increased risk persisted among those under 65 years of age who lacked common risk factors, such as obesity or diabetes (91).

The mechanisms underlying the association between COVID-19 and the development of cardiovascular diseases in the post-acute phase of the illness remain not fully elucidated.

**Figure 2:**
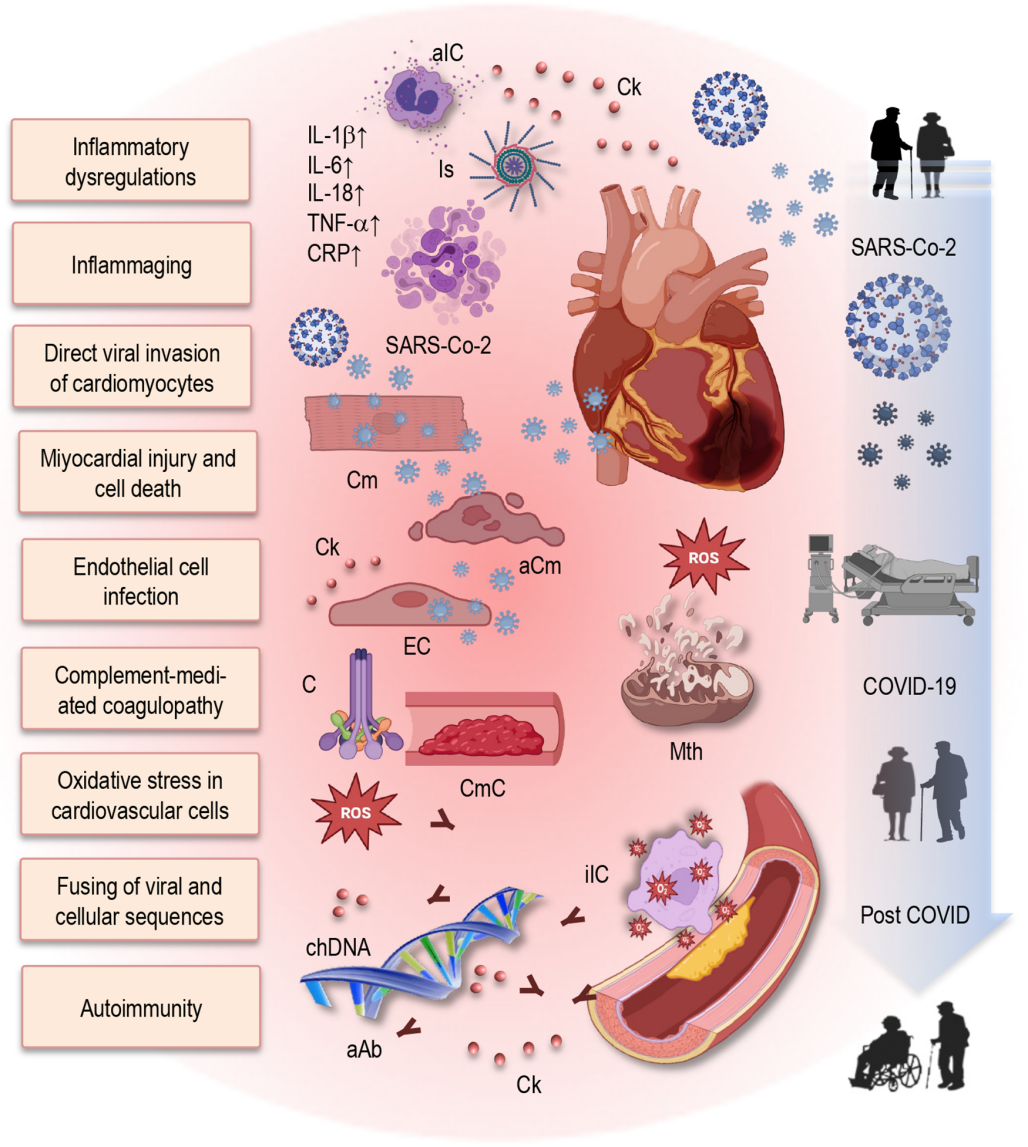
Putative mechanisms of post-COVID cardiovascular dysfunction and role of immunosenescence and inflammaging in Long COVID. This figure provides a simplified illustration of the complex interplay between inflammatory dysregulation, immune impairments, and direct viral effects contributing to cardiovascular dysfunction in post-COVID individuals. Additionally, it emphasizes the role of immunosenescence and inflammaging in exacerbating Long COVID symptoms and prolonging cardiovascular complications. Elevated levels of pro-inflammatory cytokines contribute to a persistent inflammatory state. Furthermore, SARS-CoV-2 can directly invade cardiomyocytes, leading to myocardial injury and cell death. The virus infects endothelial cells, disrupting vascular integrity and function. Increased oxidative stress, mitochondrial dysfunction, and impaired energy production in cardiovascular cells promotes tissue injury and atherosclerosis. Additionally, complement-mediated coagulopathy and microangiopathy exacerbate cardiovascular damage. Integration of the SARS-CoV-2 genome into the DNA of infected human cells may result in the expression of chimeric transcripts, potentially contributing to the continued activation of the immune-inflammatory-procoagulant cascade. Autoimmunity, including the production of autoantibodies, further damages cardiovascular tissues. aIC, activated immune cells; Ck, cytokine; Is, inflammasome; IL, interleukin; TNF, tumor necrosis factor; CRP, C-reactive protein; Cm, cardiomyocytes; aCm, apoptotic cardiomyocytes; EC, endothelial cells; C, complement; CmC, complement-mediated coagulopathy; SARS-CoV-2, severe acute respiratory syndrome coronavirus type 2; chDNA, chimeric DNA; aAb, autoantibodies; COVID-19, coronavirus disease 2019.

In the complex interplay between various factors, inflammatory dysregulations and age-related immune impairments emerge as pivotal contributors, exerting a significant influence on the development of Long COVID conditions (Figure 2). The present understanding of the pathophysiology that underlies numerous cardiovascular complications in Long COVID can be categorized into those related to immune dysregulation and inflammation, endothelial dysfunction, microvascular injury, and dysfunction in neurological signaling (61, 89, 90). However, upon closer examination, it becomes evident that inflammaging and immunosenescence may play a central role in all these categories.

Exploring mechanistic pathways provides insight into the diverse range of cardiovascular sequelae observed in the post-acute phase of COVID-19. Various mechanisms may contribute to the spectrum of cardiovascular conditions seen in individuals recovering from COVID-19 (Figure 2). One such mechanism involves the residual damage from direct viral invasion of cardiomyocytes, leading to cell death (92–95). Upon infection, SARS-CoV-2 enters cardiomyocytes through interactions with angiotensin-converting enzyme 2 (ACE2) receptors. The spike (S) protein of the virus binds to ACE2 on the surface of host cells, facilitating viral entry and subsequent infection (87, 96). This interaction between the virus and ACE2 has significant implications for viral pathogenesis and disease progression. Inside the cardiomyocyte, the virus hijacks cellular machinery to replicate, inducing cellular stress and triggering a cascade of events that cause cardiomyocyte injury and death (61, 90, 97). The presence of viral components activates innate immune responses, including the release of pro-inflammatory cytokines and chemokines, leading to inflammation and immune cell infiltration (61, 90). This inflammatory response exacerbates cellular damage, promoting cardiomyocyte apoptosis, necrosis, or pyroptosis.

Remaining damage of endothelial cells after viral infection can lead to endothelial dysfunction, inflammation, and microthrombi formation, impairing myocardial perfusion and exacerbating cardiomyocyte injury and death. Moreover, the viral infection disrupts cellular homeostasis and metabolic processes within the cardiomyocyte, leading to oxidative stress, mitochondrial dysfunction, and impaired energy production (61). The dysregulation of calcium homeostasis and contractile function may also occur, contributing to myocardial dysfunction and eventual cell death. Thus, endothelial cell infection and endotheliitis, together with transcriptional alterations affecting multiple cell types within the heart tissue, may play significant roles (98).

Furthermore, complement activation and complement-mediated coagulopathy, microangiopathy, the downregulation of ACE2, and dysregulation of the renin–angiotensin–aldosterone system (RAAS) may contribute to the complex web of potential causative factors (92–95). Complement activation (Figure 2) may occur when the immune system recognizes and responds to viral particles, triggering a cascade of events that lead to inflammation, tissue damage, and immune cell recruitment. In Long COVID, dysregulated complement activation may result in excessive inflammation and tissue injury, particularly within the cardiovascular system (95). Additionally, complement-mediated coagulopathy refers to abnormal blood clotting processes triggered by complement activation (Figure 2). This dysregulation can lead to microthrombi formation and microangiopathy, impairing blood flow and tissue perfusion, particularly in small blood vessels. In Long COVID, complement-mediated coagulopathy may contribute to the development of thrombotic events and microvascular dysfunction, exacerbating cardiovascular complications.

In addition to its role in regulating blood pressure and vascular tone, ACE2 plays a crucial role in counterbalancing the effects of ACE, another enzyme in the RAAS pathway. ACE converts angiotensin I (Ang I) into Ang II, which exerts vasoconstrictive, pro-inflammatory, and pro-fibrotic effects. Therefore, downregulation of ACE2, a key regulatory enzyme of the RAAS pathway, may represent another potential mechanism implicated in Long COVID (99, 100). Downregulation of ACE2 expression may occur as a result of viral infection or inflammatory responses, disrupting the balance of the RAAS. This dysregulation can lead to increased Ang II levels, promoting vasoconstriction, inflammation, oxidative stress, and fibrosis—all of which contribute to cardiovascular dysfunction and remodeling seen in Long COVID. Additionally, dysregulation of the RAAS caused by viral infection may lead to systemic effects on blood pressure regulation, fluid balance, and vascular function, contributing to the development and progression of cardiovascular complications in Long COVID (101).

Furthermore, the integration of the SARS-CoV-2 genome into the DNA of infected human cells has been hypothesized as a distinctive mechanism (102). This process may result in the expression of chimeric transcripts, fusing viral sequences with cellular ones, thereby potentially contributing to the continued activation of the immune-inflammatory-procoagulant cascade (Figure 2).

Autonomic dysfunction, elevated levels of pro-inflammatory cytokines, and the activation of the transforming growth factor-beta (TGF-β) signaling through the Smad pathway further exacerbate the post-acute cardiovascular complications, inducing fibrosis and the scarring of cardiac tissue (103). Under normal conditions, this signaling pathway contributes to tissue remodeling and repair processes but can also lead to pathological changes when dysregulated. In the context of post-acute COVID-19 complications, TGF-β signaling may exacerbate fibrosis and scarring of cardiac tissue, impairing heart function. Additionally, it may contribute to inflammation within the cardiovascular system, exacerbating myocarditis and promoting vascular dysfunction (7, 12, 16). Other potential post-acute cardiovascular complications include arrhythmias, thrombosis, heart failure, and dysautonomia, each of which can significantly impact cardiovascular health and increase the risk of adverse outcomes (87, 89).

Beyond these factors, aberrant persistent hyperactivated immune responses, autoimmunity, or the persistence of the virus in immune-privileged sites, (where the immune response is restrained allowing viruses to evade detection and establish persistent infections) have been suggested as potential explanations for the extrapulmonary, including cardiovascular, post-acute sequelae of COVID-19 (90, 95, 104).

Thus, the complex interplay of these diverse mechanisms sheds light on the multidimensional nature of post-acute cardiovascular complications following COVID-19 recovery. These mechanisms highlight the multifaceted nature of Long COVID pathophysiology and underline the importance of wide-ranging management strategies targeting multiple pathways to mitigate cardiovascular sequelae in affected individuals. The consequences of COVID-19 on the cardiovascular system, especially in the elderly, further underscore the need for a comprehensive understanding of the interplay between aging, immune function, and inflammatory processes to enable the development of targeted strategies for managing and preventing cardiovascular complications in this vulnerable population.

## 6 Conclusions

In summary, the interplay of inflammaging, immunosenescence, and cardiovascular aging shapes a complex landscape that significantly impacts the cardiovascular health in the elderly. Understanding the intricate molecular and cellular mechanisms involved—such as chronic inflammatory pathways and immune system alterations­—offers valuable insights into aging-related vulnerabilities. The reciprocal relationship between immunosenescence and inflammaging creates a self-reinforcing cycle, hence intensifying cardiovascular risks. Recognizing and targeting these interactions present opportunities for potential therapeutic interventions to break this cycle and alleviate the cardiovascular dysfunction in aging individuals.

The emergence of Long COVID adds another layer of complexity, thus emphasizing the role of inflammatory dysregulation and age-related immune impairments in persistent cardiovascular complications. The elevated incidence of these persistent complications represents a considerable public health challenge, thus necessitating well-designed studies to monitor individuals with a post-acute COVID-19 syndrome. Initiation of such studies is crucial, hence emphasizing the urgency in comprehending and addressing the long-term implications of the Long COVID syndrome.

Thus, our mini-review modestly contributes to the ongoing exploration of the intricate interactions in immunosenescence, inflammation, and cardiovascular aging. Considered a stepping stone in a broader journey, we hope for this concise overview to inspire further research, thus fostering innovative interventions and ultimately enhancing our comprehension and management of these conditions in the aging context.
